# Arthroscopic tri-pulley Technology reduction and internal fixation of pediatric Tibial Eminence fracture: a retrospective analysis

**DOI:** 10.1186/s12891-020-03421-z

**Published:** 2020-06-29

**Authors:** Liang Zhang, Li Zhang, Jiang Zheng, Bo Ren, Xin Kang, Xian Zhang, Xiaoqian Dang

**Affiliations:** 1 Sport Medicine Center, Honghui Hospital Affiliated with the School of Medicine, Xi’an Jiaotong University, Xi’an, 710054 Shaanxi China; 2 Anesthesiology Department, Honghui Hospital Affiliated with the School of Medicine, Xi’an Jiaotong University, Xi’an, 710054 Shaanxi China; 3 First Department of Orthopedics, The Second Affiliated Hospital of Xi’an Jiaotong University, Xi’an, 710000 China

**Keywords:** Tibial fractures, Arthroscopy, Bone development, Child, Fracture fixation

## Abstract

**Background:**

Fixing a tibial eminence fracture with a tri-pulley is a new technique. The purpose of this study was to present the early clinical outcome of arthroscopic tri-pulley suture fixation for tibial eminence fractures in children.

**Methods:**

Twenty-one pediatric patients with type II or type III anterior tibial eminence fractures were included in this retrospective study. All Patients underwent surgical fixation by tri-pulley technology and were followed up for at least 24 months. They were evaluated preoperatively and postoperatively by physical, X-ray, and computed tomography (CT) examination and subjectively with the International Knee Documentation Committee (IKDC), and Lysholm questionnaires.

**Results:**

The patients included 12 males and 9 females; mean age, 12.5 years (range, 8 ~ 16 years). They were followed-up for a median of 27 months (range, 24 ~ 39 months). We did not find post-operative instability in any of the patients by physical examination. The KT-2000 difference of both knees decreased from 9.3 ± 1.2 mm preoperatively to 2.6 ± 0.8 mm 24 months postoperatively (*P* < 0.001); the IKDC subjective knee evaluation score improved from 43.1 ± 13.2 preoperatively to 83.8 ± 6.3 postoperatively (P < 0.001); and Lysholm improved from 48.3 ± 6.21 to 87.1 ± 9.8 (P < 0.001). No unhealed fractures or epiphyseal damage were reported in the postoperative X-ray and CT.

**Conclusions:**

Arthroscopic tri-pulley fixation technology may provide a suitable technique for repair of tibial eminence fractures in skeletally immature patients.

**Level of evidence:**

Case series; Level of evidence IV.

## Background

The tibial eminence avulsion fracture has a higher incidence (3/100,000) in skeletally immature patients because the intercondylar eminence which is not fully calcified can be exposed to injuries more frequently than the ACL itsel f[1]. Because displaced fractures may cause nonunion or malunion as well as loss of knee extension or instability, types II, III, and IV are indicated for surgical intervention [2] Obviously, these modalities clearly differ from the treatment of an adult ACL injury, which is treated universally with reconstruction of the ligament as the surgical option [3] Prior studies [4–7] have demonstrated many fixation methods of the fragments of avulsion of tibial eminence, including metal hollow screws, utilizing Kirschner wires, cerclage wires, interosseus sutures, washer screws placed through the fragment, and screws and bone anchors inserted retrogradely.

Recently, arthroscopic-assisted fixation has become increasingly recommended for surgical management of intra-articular fractures [8] However, considering the main difference in tibial eminence fractures between pediatric patients and adults is the epiphysis, so far, there has been no method to provide desirable fixation of the fracture without interfering with the epiphysis. A suture anchor is a traditional and popular method of fixation that has recently been used for pediatric tibial eminence fractures, [9, 10] and double-pulley technology had been used in various orthopedic techniques [11–13] While fixing a tibial eminence fracture with a tri-pulley is a new technique.

We hypothesized that arthroscopic tri-pulley fixation would achieve a good clinical outcome with no damage to the epiphysis. Therefore, the purpose of this study was to present the use of tri-pulley technology for arthroscopic suture fixation for tibial eminence fractures in pediatric patients and particularly to assess its early clinical outcome.

## Methods

### Patients

This was a retrospective analysis of the data from 21 skeletally immature patients with tibial eminence avulsion fracture who were treated in the Sports medicine department of HongHui hospital affiliated to Xi An JiaoTong University from January 2014 to December 2015. Tibial eminence fractures were defined according to the classification of Meyers and McKeever modified by Zaricznyj [14, 15]

The inclusion criteria were:1) aged <16 years; 2) displaced ACL tibial avulsion fractures (Meyers and McKeever types II, II I[14]) confirmed by plain radiography (antero-posterior and lateral views), magnetic resonance imaging (MRI), or computed tomography (CT) scan; 3) underwent arthroscopic tri-pulley fixation. The exclusion criteria included: 1) patients with associated tibial plateau or femur fracture, neurovascular injury, or knee dislocation; 2) patients who had a history of osteoarthritis, previous surgery or infection.

This study was approved by the local ethics committee of HongHui hospital affiliated to Xi An JiaoTong university (No. YDYX20140121) and the requirement for informed consent was waived.

### Surgical technique

**Fig. 1:**
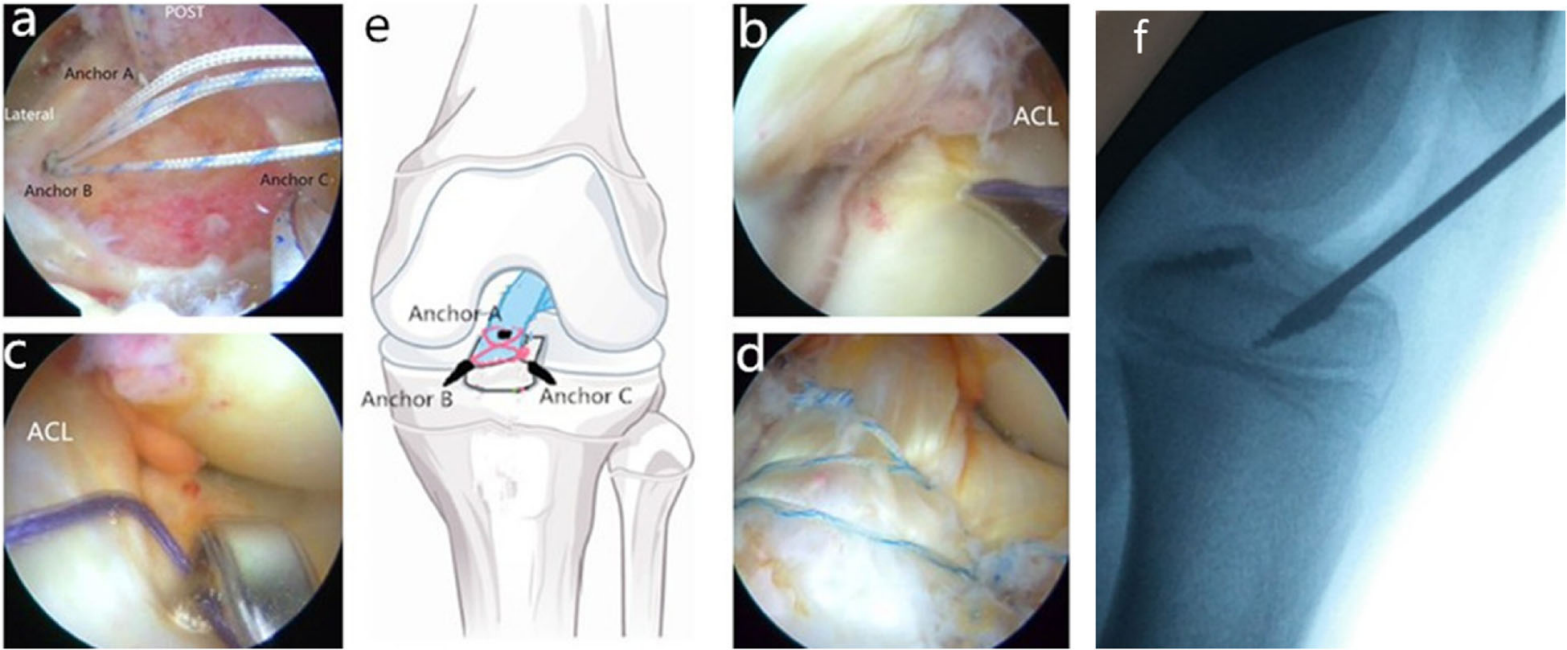
Tri-pulley technology. **a** Three anchors were placed into the edge of the fracture bed. Post edge (anchor A), anterolateral edge (anchor B) and anteromedial edge (anchor C). **b & c** A hook penetrated the distal part of the anterior cruciate ligament (ACL) to thread through anchor A from different sides of the ACL. **d** Image after fracture reduction and fixation by tri-pulley technology. **e** Schematic drawing of the tri-pulley technology. **f** X-ray in the operation room ensured the anchor was on the proximal epiphyseal

**Fig. 2:**
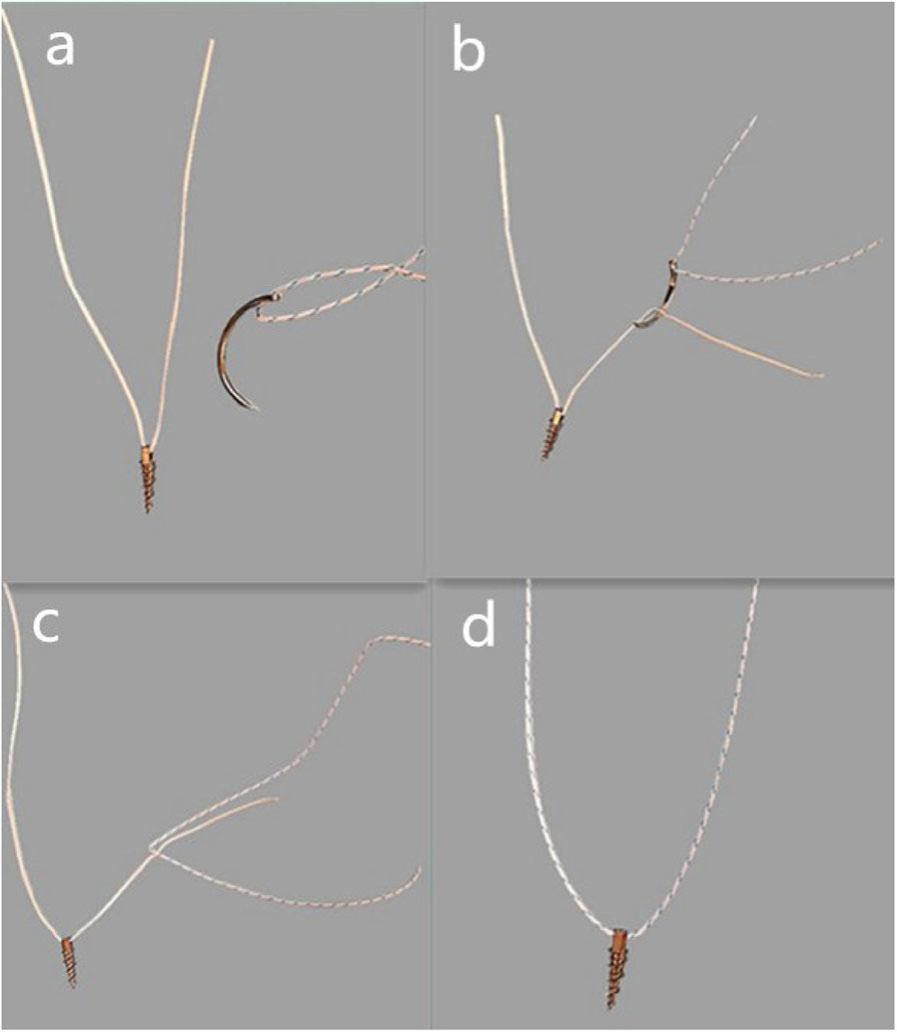
Pass Technology **a** Anchor A with a white thread and a needle with colored thread form another anchor. **b** The needle was passed through the white thread. **c** The needle was moved off and the white thread was pulled to thread the colored thread through anchor A. **d** Anchor A was combined with another anchor by the colored thread

**Fig. 3:**
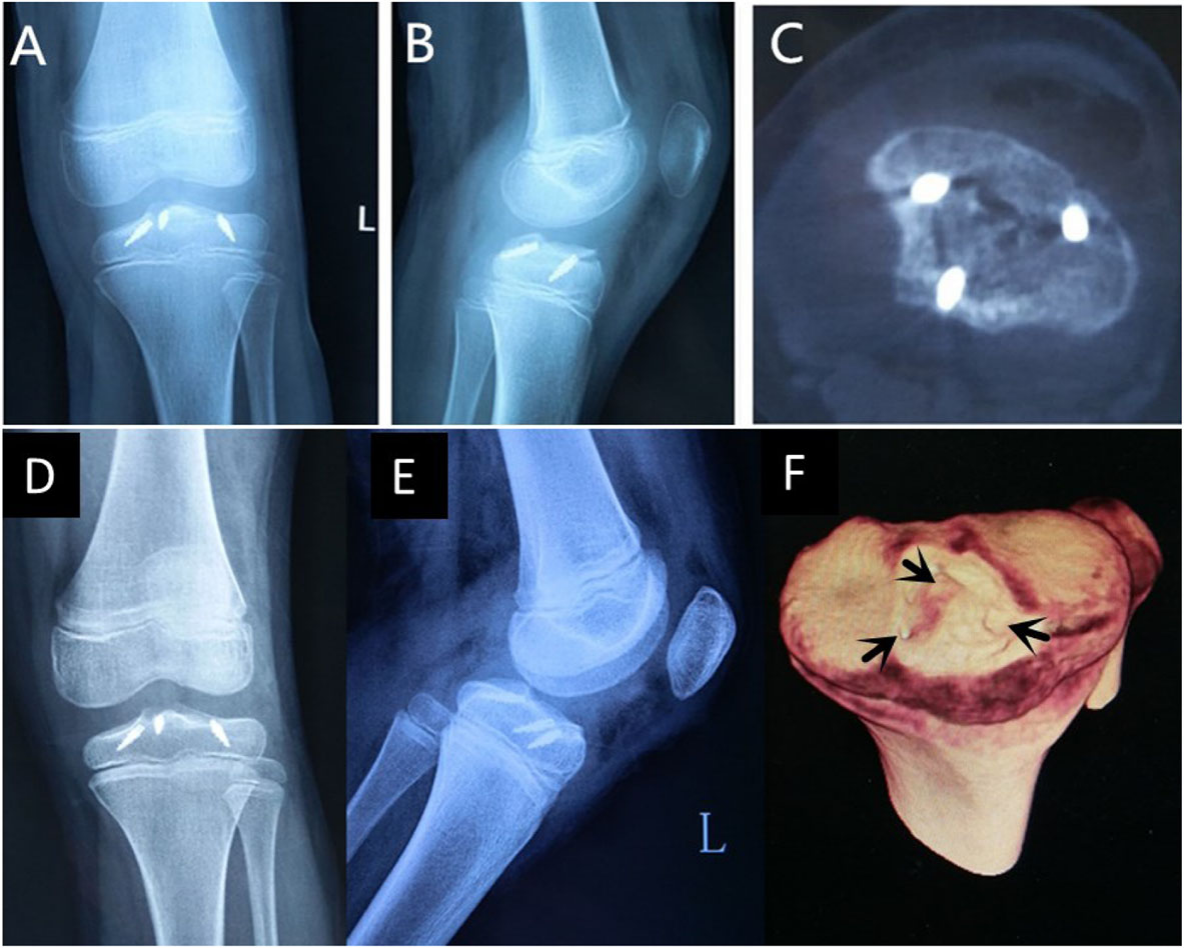
Post-operation images. **a**: Conventional radiography anterior posterior (AP) and **b**: lateral view and **c**: computed tomography (CT) scan immediately after surgery. **d**: Conventional radiography AP and **e:** lateral view and **f**: CT scan 6-months post-operation. Showing good epiphyseal union of the fracture

All patients were operated on by two surgeons who had more than 10 years of experience in using the tri-pulley surgical technique. Under general anesthesia, the patients were placed in the supine position and knee 90°flexion. The method of surgery was presented in Fig. 1. The tourniquet pressure was set to reach a 50-mmHg higher pressure than systolic blood pressure. Standard anteromedial (AM) and anterolateral (AL) portals were performed to wash out the hemarthrosis and examine the joint. Because all cases were acute, the first stage of the procedure was evacuation of the hematoma. Accompanying injuries were examined and treated when necessary. The soft tissue was debrided with a shaver to enhance visualization. Fibrin clots and small fracture fragments were removed underneath the main fragment and from the tibial crater if needed. The anterior and transverse horns of the menisci were probed carefully, because if the menisci interposed into the fracture this would affect fracture reduction. A shaver was inserted from the AM portal to prepare the fracture bed by clearing the debris. Attention was paid to protect the intermeniscal ligament and anterior horn of the meniscus. Then, the first anchor (Smith & Nephew TWINFIX Ti 2.8 HS Anchor, US) was inserted into the trailing edge of the fracture bed (anchor A) from the AM portal under view from the AL portal (Fig. 1). The thickness between the point where we wanted to place the anchors and the growth plate was measured and planned pre-operatively by CT scan to ensure there was enough room for the anchor. The anchor was tilted 45 degrees in the sagittal axis with axial alignment of the lower extremity. The second anchor (anchor B) was inserted into the anterolateral edge of the fracture bed from the AM portal under view from the AL portal at 45 degrees to the coronal plane with axial alignment to the lower extremity. The third anchor (anchor C) was inserted into the anteromedial edge of the fracture bed from the AL portal under view from the AM portal at 45 degrees in the coronal plane with axial alignment to the lower extremity. The positioning of the anchors was carefully monitored by X-ray (Fig. 1f). A hook penetrated the distal part of the ACL to thread through anchor A from different sides of the ACL (Fig. 1b, c). The two ends of the thread crossed in front of the ACL, one end was tied with the suture of anchor B to form a double pulley system; the other end of the anchor A passed through anchor C by suture (Ethicon, Somerville, NJ) pass technology (Fig. 2). In this way the tri-pulley system was completed (Fig. 1d, e). Then arthroscopy-guided fracture reduction was performed using the probe. The suture was tightened to form a network structure on the surface of the fracture. The threads were knotted to fix the fracture with the knee at 30°flexion under the stress of the posterior drawer. Impingement, ACL tension and fracture reduction were examined to check the fracture reduction. The operation was finished by a thorough lavage and insertion of a drain. The positions of the anchors were confirmed postoperatively with conventional radiography (AP and lateral views) and CT scan (Fig. 3a, 4b, 4c).

### Rehabilitation

After operation, the knee was placed in a hinged knee brace locked in extension for 2 weeks. Ankle pumps, quad sets and straight leg raise movements were allowed as soon as possible after operation. Two weeks later, physiotherapy was started involving both active and passive exercises twice a day and partial weight bearing was allowed. The brace was then adjusted to allow increased range of motion of the knee. Patients were initially allowed partial weight bearing with crutches, and at 4 weeks, full weight bearing was allowed. Isometric quadriceps muscle exercises were performed throughout the immobilization period. Return to sports was permitted at 6 months postoperatively, after knee stability, range of motion, muscle strength, and proprioception were restored.

### Clinical evaluation

Every patient was evaluated preoperatively on the basis of clinical examination, MRI and CT scans. The objective was to assess the presence of nonunion, displacement, and epiphyseal premature closure of fractures. The clinical evaluation was mainly on knee function.

### Outcomes and follow-up

The patients were followed up at 1.5 months, 3 months, 6 months, and 12 months and then yearly. All of these examinations were performed by two senior sports medicine specialists. In case of doubt, a third senior sports medicine specialist was invited to re-evaluate and obtain a comprehensive opinion. Anteroposterior laxity was assessed with the Lachman, anterior drawer and pivot-shift test s[16, 17]. The tests were graded normal (−); mild (+); moderate (++); and severe (+++). The KT-2000 test (MEDmetric Corp San Diego, CA) was used for quantification of anterior tibial translation. Knee range of motion was evaluated actively and passively with a goniometer. Anteroposterior and lateral radiographs were obtained 1.5 and 3 months postoperatively to assess fracture healing. A fracture was considered united if no fracture line was visible radiographically at 3 months.

A comprehensive clinical examination was performed. Knee function was evaluated by Lysholm functional and the IKDC (International Knee Documentation Committee) scores [18, 19] and ROM. At final follow-up, all patients were reviewed by an independent orthopedic surgeon uninvolved with their care. X-ray and CT images were examined 6-months postoperatively to evaluate fracture healing and epiphysis development.

### Statistical analysis

All statistical analyses were performed with SPSS 13.0 software (SPSS, Chicago, IL, USA). Non-normally distributed continuous data are expressed as medians (range or IQR). Categorical data are expressed as n (%). The chi-square test was used to compare the preoperative and postoperative Lachman, anterior drawer and pivot-shift tests. A t test was used to compare the preoperative and postoperative Lysholm scores, IKDC scores, and KT-2000 test. Statistical analysis was conducted by an independent statistician to surgical outcomes. P<0.05 was considered statistically significant.

## Results

### Baseline data

The study population consisted of 12 male (57.1%) and 9 female (42.9%) patients. The mean patient age was 12.5 years (range, 8 to 16 years). The cause of injury was related to sports in all cases. On the basis of the Meyers and McKeever system, the fractures included Type II (14 (66.7%) patients), Type III/A (3 (14.3%) patients) and Type III/B (4 (19.0%) patients). The mean time from injury to surgery was 4 days (range, 2 to 14 days). The time taken for the surgery ranged from 42 to 77 min. The demographic and clinical data of the patients are presented in Table 1.

**Table 1:** Baseline and clinical data from the 21 patients

Mean Age, years	Sex male/female	Fracture Type, numbers	Time to surgery after injury, days	Length of operation, min	Length of stay, days	Follow-up, months
12.7 ± 2.1	14 /7	Type II 15	7.7 ± 1.7	59.4 ± 8.3	6.3 ± 1.2	28.4 ± 5.6
		Type III 6				

### Clinical outcomes

Six months after the operation fracture healing and epiphysis development was monitored by X-ray and CT imaging. No cases of epiphyseal closure and fracture nonunion occurred in this group (Fig. 3d, e, f).

Prior to surgery, 18 patients (85.7%) were graded ++ or +++ according to Lachman score. The anterior drawer examinations showed 15 cases (71.4%) were ++ or +++. While pivot shift test showed only one patient of ++ grade and no patient with +++. The follow-up examinations performed after six-month follow-up demonstrated one case of instability (Lachman + and anterior drawer + but pivot shift -, Table 2).

**Table 2:** Comparison of fracture stability prior to treatment with 1-year follow-up

Follow up	Pre-op				Post-op (12 months)				*P* Value
	–	+	++	+++	–	+	++	+++	
Lachman (numbers of patient)	0	3	12	6	20	1	0	0	.001
Anterior drawer (numbers of patient)	0	6	12	3	20	1	0	0	.001
Pivot shift (numbers of patient)	9	10	1	0	21	0	0	0	.002

The tests were graded normal (−); mild (+); moderate (++); and severe (+++)

*Op* operation

The functional outcome according to the KT-2000 showed the difference between the two knees decreased from 9.3 ± 1.2 mm preoperatively to 2.6 ± 0.8 mm postoperatively at 1-year (*P* < 0.001); the IKDC subjective knee evaluation score improved from 43.1 ± 13.2 to 83.8 ± 6.3 (*P* < 0.001); and Lysholm score improved from 48.3 ± 6.21 to 87.1 ± 9.8 (*P* < 0.001); The range of motion was improved from 53.5 ± 5.7° preoperative to 134.2 ± 7.1° postoperative at 1-year (*P* < 0.001); The flexion angle was improved from 89.4 ± 4.9° preoperative to 135.1 ± 6.8° postoperative at 1-year (*P* < 0.001); The extension angle was improved from 35.9 ± 6.1° preoperative to 0.9 ± 0.3° postoperative at 1-year (*P* < 0.001); all of the improvements were statistically significant .

There were no adverse events such as nonunion of bone, failure of internal fixation and epiphyseal premature closure.

## Discussion

The aim of this study was to present the use of tri-pulley technology for arthroscopic suture fixation for tibial eminence fractures in pediatric patients and to assess its early clinical outcome. Twenty-one pediatric patients who underwent the procedure showed no cases of epiphyseal closure and fracture nonunion. The KT-2000, the IKDC and Lysholm score all were improved (*P* < 0.001). There were no adverse events. There was still a 2.6 mm difference between the two sides in KT-2000, which was related to the relative relaxation of the ligaments around the knee joint in children.

Other studies have presented different methods of arthroscopy-assisted reduction and fixation of the displaced tibial eminence fractures in pediatric patients and have shown good outcome s[10, 20]. However, a comparison between open reduction and internal fixation or arthroscopic reduction and internal fixation suggested that arthroscopic reduction and internal fixation extended operation times from 98 to 141 mi n[21]. This was not seen in this study where operation times ranged from 42 to 77 min, all shorter than mean time for open reduction performed in that study. Though there may be differences in the way the surgery time is measured and the experience of the surgeons we think this may reflect the most important finding of the present study; that the unique tri-pulley technology fixation technology was simple and reliable and is suitable for skeletally immature patients.

The tibial eminence avulsion fracture is a severe injury of the knee joint, and most of them happen mainly in childhood or at a young ag e[22]. There are many methods described for treatment of tibial eminence avulsion fracture, including either arthrotomy or arthroscopy. Screw fixation has been reported as being successful [23] but has disadvantages. It cannot completely avoid damaging the epiphysis and removal of the screws is necessary, especially for metal screws. Incorrect positioning of a screw can lead to impingement and articular cartilage damage. Screws generally are not an option for small fracture fragments, because of the inherent risk of fracturing the fragment. Absorbable suture fixation performed under arthroscopy has also been reported with good results [24] . But this method requires two 2.4-mm holes to be drilled through the proximal tibia into the joint, Which could not completely avoid damaging the epiphyseal. All patients who were treated by arthroscopic reduction and internal stabilization with absorbable pins, achieved full range of motion and radiological fracture consolidations [25] But as wells as the potential damage to the epiphyseal plate, the strength of fixation and absorption time must be considered. Treatment of tibial eminence fractures in children must consider two aspects: the perfect reduction and fixation, and avoiding injury of the epiphysis. Kim [26] reported an epiphyseal-sparing fixation by using one anchor in immature patients. But fixation with a single anchor could not provide sufficient rotational stability. In et al [6, 7] reported using suture anchors with good results in a comminuted case and in a skeletally immature patient. But in those studies, each anchor is independent and does not contribute to a network. The advantages of using a tri-pulley method were:1) Three independent anchors can be connected into a system to form a network structure on the surface of the fracture block, which can more evenly fix the bone block, avoid stress concentration, and have a better compression effect on the bone block, which is conducive to fracture healing; 2) When the three anchors are integrated, the stress of each anchor is more dispersed during the action of the anterior fork ligament against the bone mass, reducing the risk of internal fixation failure.

Here we have demonstrated that arthroscopic tri-pulley technology fixation is a promising surgical method for displaced tibial eminence avulsion fractures in pediatric patients. The most important advantage of this technology is it will cause no damage to the epiphyseal plate, which has been confirmed in our postoperative follow-up results. We placed the anchor with a tilt of 45 degrees with axial alignment of the lower extremity. In that way, it can further reduce the risk of epiphyseal injury. Furthermore, after the anchors were embedded, X-ray assessment ensured that the anchor was on the proximal epiphyseal. Secondly, the tri-pulley fixation technique as a closed-loop structure mechanical system that can provide three-point reticular fixation, which can effectively stabilize the fracture. So the patients could begin functional exercise as soon as possible, which is beneficial to the recovery of the knee joint function [27] Thirdly, It does not cause any mechanical problems in function of the knee joint, because there are only two knots in the tri-pully fixation systerm. Although it is impossible for the patient to achieve complete reduction of the bone block, the bone is reduced and fixed in the bed by the tri-pulley fixation technique only 1-2 mm bones protrude left, which were confirmed under arthroscope in this group of cases. So, we think it provides a more rigid and anatomic fixation than other techniques. Due to less interference of internal fixation and accurate reduction of fracture, the recovery of extension function is effectively guaranteed, No patients with extension restrictions were found in this study, and the extension angle was improved from 35.9 ± 6.1° preoperative to 0.9 ± 0.3° postoperative at 1-year. Fourthly, Compared to screw fixation, tri-pulley fixation is unlikely to break the bone fragment, so it can be used for small or comminuted fragment. Finally, removal of the anchor is generally not required, so there is no need for a second surgical procedure and so this arthroscopic method also causes a small amount of trauma.

In this study, metal anchors were used instead of absorbable anchors, this raises three important points: 1) As metal anchors are clearly visible under X-ray, the relationship between the anchors and epiphyses can be effectively evaluated during surgery. 2) Since the anchors need to be placed into the bone bed, which is cancellous bone, and metal anchors do not require bone holes to be prepared in advance, there is less damage to the cancellous bone, so the metal anchors are simple to operate and secure 3) Because metal anchors are not absorbable, whether they affect the epiphyses or not still needs to be evaluated. No cases of epiphyseal influence were found in the follow-up of this study, but long-term follow-up results are needed. Therefore, absorbable anchors can be used on the premise that the epiphysis will not be damaged in the effective evaluation, and to reduce the possibility of later interference with the epiphysis.

This study has some limitations. The first limitation is the small number of cases compared with other reports related to the injury. A larger study is now required to see if these results are repeated in more subjects. There was no comparison between this method and another more traditional open reduction and fixation or screw fixation methods. A randomized controlled trial would reveal whether this method is really more effective than other methods. Another limitation is that there are no biomechanical results to test the fixation strength during this study. Finally, it is necessary to have longer observation and follow-up to demonstrate the long-term efficacy of tri-pulley treatment in pediatric tibial eminence fracture.

## Conclusions

In conclusion, arthroscopic tri-pulley fixation technology is a simple and reliable technique that can protect the epiphysis of skeletally immature patients. It provided clinical and radiological improvements and preserved the function of the injured knee in pediatric patients.

## Data Availability

The datasets used and/or analyzed during the current study are available from the corresponding author on reasonable request.

## Abbreviations

CT: Computed tomography
IKDC: International Knee Documentation Committee
ACL: Anterior cruciate ligament
MRI: Magnetic resonance imaging
AM: Anteromedial
AL: Anterolateral
